# Dyskinesia in an unmedicated adolescent with a 5-year history of tobacco use: a case report

**DOI:** 10.1186/s13256-016-1021-5

**Published:** 2016-08-16

**Authors:** Anthony A. Olashore, Oluyemi O. Akanni, Philip R. Opondo

**Affiliations:** 1 Department of Psychiatry, University of Botswana Medical School, Gaborone, Botswana; 2 Forensic unit, Federal Neuropsychiatric Hospital, Benin City, Edo State Nigeria

**Keywords:** Dyskinesia, Unmedicated, Adolescent, Tobacco use, Case report

## Abstract

**Background:**

Tobacco use among mentally ill patients is approximately two to three times higher than that in the general population. Withdrawal from tobacco is a common occurrence and many authors have described various symptoms of nicotine withdrawal. Dyskinesia, however, has not been reported as one of the symptoms of nicotine withdrawal in adolescents without any psychopathology or medical illness.

**Case presentation:**

We report a case of a 17-year-old Motswana boy of the Tswana ethnic group with primary school education and a 5-year history of tobacco use who developed dyskinesia approximately 48 hours after cessation of tobacco and had some relief with nicotine gum.

**Conclusion:**

In addition to the documented symptoms of nicotine withdrawal, clinicians should look out for dyskinesia, which may be one of the rare symptoms of withdrawal in chronic tobacco users.

## Background

Tobacco use among mentally ill patients is approximately two to three times higher than that in the general population [1, 2]. While some use this substance to self-medicate symptoms of psychosis, others use it to reduce the side effect of neuroleptics such as neuroleptic-induced dyskinesia [1–3]. Withdrawal from tobacco is a common occurrence, and several authors have described various symptoms of nicotine withdrawal, such as restlessness, anxiety, insomnia, dysphoria, tension, and even delirium in the mentally ill [4–7]. Hypomania has also been reported during nicotine cessation treatment with bupropion [8]. Dyskinesia has not been reported as one of the symptoms of nicotine withdrawal, especially in unmedicated adolescents without any psychopathology or medical illness to the best of the authors’ knowledge, hence the need to report this case.

## Case presentation

A 17-year-old Motswana boy of the Tswana ethnic group with a primary school education was brought to our psychiatric hospital with a 5-year history of tobacco smoking and delinquent behaviors. He is the first in a family of three children; both his parents are alive but separated. He lives with his mother who is of low socioeconomic status; she has a busy schedule and works full time. There is no history of mental illness or substance abuse in the family. He was observed at the age of 11 years to exhibit conduct disorder behaviors such as disobedience, stealing, truancy, and hanging out with “street kids.” He was first introduced to tobacco smoking by his friends at age 12 years. He started with a Peter Stuyvesant (nicotine content of 1.3 mg/cigarette) brand of cigarette which was initially unpleasant; however, he continued with persuasion from his friends. He gradually stepped up his use from one cigarette/day over the next 2 to 3 years to approximately 20 to 30 cigarettes per day to sustain the relaxing and stimulating effect which improved his daily performance. He admitted to craving for this substance to the extent of doing dirty jobs for people to sustain the habit and neglecting other previous forms of enjoyment, such as watching television with family members. He has had several unsuccessful attempts at controlling the amount he took in a day despite the knowledge of its harmful consequences. His longest period of abstinence was 3 months in a rehabilitation center which was approximately 3.5 years ago. He had once experimented with cannabis and alcohol, but he never enjoyed these substances and so did not continue.

Two months prior to his index presentation at our hospital, he progressively neglected his personal hygiene and food, became emaciated, and spent more time cigarette smoking (that is, smoking continuously); he decided to seek medical attention at this time.

There were no psychotic symptoms on admission, but he was very restless, irritable, and had intense craving for this substance. In addition he complained of headache and insomnia.

He has had no previous treatment or admission for any psychological disorder and was never on any psychotropic medication before his index presentation at our hospital. A year after he started smoking cigarettes, his mother decided to seek spiritual help when he was observed to be smoking cigarettes at the expense of other activities, pilfering, and playing truant, but there was no significant improvement. His cigarette smoking subsequently became excessive over the next 6 months and he consequently started neglecting his personal hygiene, withdrawing from family activities, and preferring to smoke cigarettes rather than eat; thus, he was becoming emaciated. As a result, his mother was advised to try a rehabilitation center. He spent 3 months in a rehabilitation center 3 years prior to his presenting at our hospital. He went through drug education, counselling, and was abstinent for only this period. He had neither psychotic nor mood symptoms before or during the period of rehabilitation, and did not experience any abnormal movement. He only complained of restlessness and tension; nevertheless, he was not placed on any medication other than multivitamins. While he was in the rehabilitation center, he was completely abstinent and his appetite and weight improved considerably. On leaving the rehabilitation center, he attended follow-up only once before he defaulted. Afterwards, he went back to smoking cigarettes and has had no period of abstinence until his index admission to our mental health facility.

Before he started smoking cigarettes, he was described as an easy child, quite cheerful, and an outgoing person who enjoyed the company of other children.

A mental state examination at his index admission to our hospital revealed agitation, but there was no abnormality of speech, thought, or perception. He described his mood as fine, but objectively it was anxious.

A physical examination revealed no significant abnormality. His blood pressure was 110/70 mmHg, pulse rate was 90 beats/minute, and his temperature was 37 °C. Investigations such as full blood count, liver function test, thyroid function test, as well as computed tomography (CT) scanning of his brain revealed no significant abnormality. Urine drug screening was negative for substances which included cannabis, cocaine, and phencyclidine, except for benzodiazepines, which was given to reduce restlessness and to improve sleep.

The working diagnosis made was mental and behavioral disorder due to psychoactive substance use; nicotine dependence with comorbid conduct disorder.

Three days postadmission, he was observed to be having some abnormal involuntary movement such as occasional chewing movements, trunk twisting, and truncal tremor. During an interview he tried to conceal the involuntary movements of his hands by holding his chair with a firm grip. According to the nurses’ reports, these movements often disappeared during sleep and briefly whenever his attention was called to them. He admitted to the fact that he first experienced these movements approximately 2 years ago and has also observed their disappearance whenever he smokes. This claim was supported following some relief which he experienced with nicotine gum (Nicorette); an offer which he previously refused.

He was on admission for 4 weeks with scheduled sessions with a psychologist on drug counselling and education. In addition he was placed on diazepam 10 mg on the first night and twice daily for 5 days in addition to nicotine gum which was made available to him on demand.

His level of hygiene as well as his appetite improved approximately 2 weeks after admission in response to therapy. In addition, his abnormal movements reduced after 3 weeks on nicotine gum after which he was discharged home with the gum. He was to continue with monthly psychological sessions on an out-patient basis since there was no formal rehabilitation program in the facility. He was seen only once on follow-up during which it was noted that he did well on nicotine gum without any adverse effect. His appetite, level of hygiene, and weight were also well maintained but he then defaulted.

## Discussion

Here we report a case of an unmedicated young man with chronic use of tobacco who developed dyskinesia approximately 48 hours after cessation of tobacco and had some relief with nicotine gum. The fact that he first experienced these abnormal movements approximately 2 years ago following tobacco cessation or reduction in his level of use and had been self-medicating with cigarettes suggests it had become a chronic condition.

It has been shown that nicotine increases striatal and cortical dopamine release in patients with schizophrenia [9]; hence, the suggestion that nicotine-induced cognitive enhancement and locomotor stimulation is dependent on dopamine release [9, 10]. With chronic exposure to nicotine, tolerance ensues leading to a reduction in the effect of nicotine, and eventually a decrease in dopamine release [11]. This may partly explain the increase in the level of use in order to sustain its cognitive-enhancing effect, even among non-psychiatric users, thus explaining one of the reasons why our index patient has been unable to stop smoking cigarettes. In addition, dyskinesia of various kinds have been extensively reported among psychiatric patients on antipsychotic treatments, due to the blockade of dopamine activities [4] and, so, dyskinesia may result consequent to a sudden reduction in nicotine use among chronic users. Dyskinesia may therefore be one of the symptoms of withdrawal in the same way that a prolonged blockade of dopamine at the dorsal striatum by antipsychotic medication causes dyskinesia. This unpleasant side effect may be another reason why it had been very difficult for him to stop tobacco use in addition to its cognitive-enhancing effect.

### Limitation

Our inability to monitor his blood nicotine level due to the unavailability of facilities limits our ability to correlate his blood nicotine level with the development and severity of his symptom (that is, dyskinesia).

## Conclusions

In addition to losing the cognitive-enhancing effect of cigarette smoking and experiencing common withdrawal effects such as restlessness, tension, and delirium, dyskinesia, which we have reported as a dreadful effect of withdrawal, may be one of the unexplored or very rare reasons why many chronic cigarette smokers find it difficult to stop the habit.

Clinicians should look out for a history of dyskinesia following withdrawal from tobacco in a chronic tobacco user.

## Abbreviation

CT, computed tomography
